# Mammographic density assessed on paired raw and processed digital images and on paired screen-film and digital images across three mammography systems

**DOI:** 10.1186/s13058-016-0787-0

**Published:** 2016-12-19

**Authors:** Anya Burton, Graham Byrnes, Jennifer Stone, Rulla M. Tamimi, John Heine, Celine Vachon, Vahit Ozmen, Ana Pereira, Maria Luisa Garmendia, Christopher Scott, John H. Hipwell, Caroline Dickens, Joachim Schüz, Mustafa Erkin Aribal, Kimberly Bertrand, Ava Kwong, Graham G. Giles, John Hopper, Beatriz Pérez Gómez, Marina Pollán, Soo-Hwang Teo, Shivaani Mariapun, Nur Aishah Mohd Taib, Martín Lajous, Ruy Lopez-Riduara, Megan Rice, Isabelle Romieu, Anath Arzee Flugelman, Giske Ursin, Samera Qureshi, Huiyan Ma, Eunjung Lee, Reza Sirous, Mehri Sirous, Jong Won Lee, Jisun Kim, Dorria Salem, Rasha Kamal, Mikael Hartman, Hui Miao, Kee-Seng Chia, Chisato Nagata, Sudhir Vinayak, Rose Ndumia, Carla H. van Gils, Johanna O. P. Wanders, Beata Peplonska, Agnieszka Bukowska, Steve Allen, Sarah Vinnicombe, Sue Moss, Anna M. Chiarelli, Linda Linton, Gertraud Maskarinec, Martin J. Yaffe, Norman F. Boyd, Isabel dos-Santos-Silva, Valerie A. McCormack

**Affiliations:** 1 Section of Environment and Radiation, International Agency for Research on Cancer, 150 cours Albert Thomas, 69372 Lyon, Cedex 09, France; 2 Centre for Genetic Origins of Health and Disease, Curtin University and the University of Western Australia, Perth, Australia; 3 Channing Division of Network Medicine, Department of Medicine, Brigham and Women’s Hospital, Harvard Medical School, Boston, MA USA; 4 Moffitt Cancer Center, Tampa, FL USA; 5 Department of Health Sciences Research, Mayo Clinic, Rochester, MN USA; 6 Department of Surgery, Istanbul Faculty of Medicine, Istanbul University, Istanbul, Turkey; 7 Institute of Nutrition and Food Technology, University of Chile, Santiago, Chile; 8 Centre for Medical Image Computing, University College London, London, UK; 9 Faculty of Health Sciences, University of the Witwatersrand, Johannesburg, South Africa; 10 Marmara University School of Medicine Department of Radiology, Istanbul, Turkey; 11 Slone Epidemiology Center, Boston University, Boston, MA USA; 12 Division of Breast Surgery, Department of Surgery, The University of Hong Kong, Hong Kong, People’s Republic of China; 13 Department of Surgery, Hong Kong Sanatorium and Hospital, Hong Kong, People’s Republic of China; 14 Cancer Epidemiology Centre, Cancer Council Victoria, Melbourne, Victoria Australia; 15 Centre for Epidemiology and Biostatistics, Melbourne School of Population and Global Health, The University of Melbourne, Melbourne, Victoria Australia; 16 Cancer Epidemiology Unit, Instituto de Salud Carlos III and CIBERESP, Madrid, Spain; 17 Breast Cancer Research Group, University Malaya Medical Centre, University Malaya, Kuala Lumpur, Malaysia; 18 Cancer Research Malaysia, Subang Jaya, Malaysia; 19 Department of Global Health and Population, Harvard T.H. Chan School of Public Health, Boston, MA USA; 20 Center for Research on Population Health, Instituto Nacional de Salud Pública, Mexico City, Mexico; 21 Section of Nutrition and Metabolism, International Agency for Research on Cancer, Lyon, France; 22 National Cancer Control Center, Haifa, Israel; 23 Cancer Registry of Norway, Oslo, Norway; 24 Department of Nutrition, Institute of Basic Medical Sciences, University of Oslo, Oslo, Norway; 25 Department of Preventive Medicine, University of Southern California, Los Angeles, CA USA; 26 Norwegian Center for Minority and Migrant Health Research (NAKMI), Oslo, Norway; 27 Department of Population Sciences, Beckman Research Institute, City of Hope, CA USA; 28 Isfahan University of Medical Sciences, Isfahan, Iran; 29 Department of Surgery, Asan Medical Center, Seoul, Republic of Korea; 30 Cairo University, Cairo, Egypt; 31 Woman Imaging Unit, Radiodiagnosis Department, Kasr El Aini, Cairo University Hospitals, Cairo, Egypt; 32 Department of Surgery, Yong Loo Lin School of Medicine, Singapore, Singapore; 33 Saw Swee Hock School of Public Health, National University of Singapore, Singapore, Singapore; 34 NUS Graduate School for Integrative Sciences and Engineering, National University of Singapore, Singapore, Singapore; 35 Gifu University, Gifu, Japan; 36 Aga Khan University Hospital, Nairobi, Kenya; 37 Julius Center for Health Sciences and Primary Care, University Medical Center Utrecht, Utrecht, The Netherlands; 38 Nofer Institute of Occupational Medicine, Łódz, Poland; 39 Department of Imaging, Royal Marsden NHS Foundation Trust, London, UK; 40 Division of Cancer Research, Ninewells Hospital & Medical School, Dundee, UK; 41 Wolfson Institute of Preventive Medicine, Queen Mary University of London, London, UK; 42 Ontario Breast Screening Program, Cancer Care Ontario, Toronto, Canada; 43 Princess Margaret Cancer Centre, Toronto, Canada; 44 University of Hawaii Cancer Center, Honolulu, HI USA; 45 Medical Biophysics, University of Toronto, Toronto, Canada; 46 Department of Non-Communicable Disease Epidemiology, London School of Hygiene & Tropical Medicine, London, UK

**Keywords:** Breast density, Image processing, Mammographic density assessment, Breast cancer, Methods

## Abstract

**Background:**

Inter-women and intra-women comparisons of mammographic density (MD) are needed in research, clinical and screening applications; however, MD measurements are influenced by mammography modality (screen film/digital) and digital image format (raw/processed). We aimed to examine differences in MD assessed on these image types.

**Methods:**

We obtained 1294 pairs of images saved in both raw and processed formats from Hologic and General Electric (GE) direct digital systems and a Fuji computed radiography (CR) system, and 128 screen-film and processed CR-digital pairs from consecutive screening rounds. Four readers performed Cumulus-based MD measurements (*n* = 3441), with each image pair read by the same reader. Multi-level models of square-root percent MD were fitted, with a random intercept for woman, to estimate processed–raw MD differences.

**Results:**

Breast area did not differ in processed images compared with that in raw images, but the percent MD was higher, due to a larger dense area (median 28.5 and 25.4 cm^2^ respectively, mean √dense area difference 0.44 cm (95% CI: 0.36, 0.52)). This difference in √dense area was significant for direct digital systems (Hologic 0.50 cm (95% CI: 0.39, 0.61), GE 0.56 cm (95% CI: 0.42, 0.69)) but not for Fuji CR (0.06 cm (95% CI: −0.10, 0.23)). Additionally, within each system, reader-specific differences varied in magnitude and direction (*p* < 0.001). Conversion equations revealed differences converged to zero with increasing dense area. MD differences between screen-film and processed digital on the subsequent screening round were consistent with expected time-related MD declines.

**Conclusions:**

MD was slightly higher when measured on processed than on raw direct digital mammograms. Comparisons of MD on these image formats should ideally control for this non-constant and reader-specific difference.

**Electronic supplementary material:**

The online version of this article (doi:10.1186/s13058-016-0787-0) contains supplementary material, which is available to authorized users.

## Background

Mammographic density (MD), a measure of the radiodense tissue in the breast, is a strong marker of breast cancer (BC) risk [1]. MD is increasingly being incorporated into BC research and clinical practice, for example in BC risk prediction models [2], as a marker for the effectiveness of therapeutic drugs mediated through MD [3], and in risk-based stratification for tailored BC screening regimens [4]. To enable these applications, estimates of differences in MD between women and within women over time are needed. However, obtaining directly comparable MD measurements is challenged by the fact that no single MD measurement tool is used universally; there are more than 10 quantitative methods currently in use [5–8]. Further, for the widely used threshold method, MD measurements are affected by well-documented reader variability [9, 10]. Less studied is the influence of the type of mammogram used for MD measurements. Images originate from a variety of imaging modalities and mammography systems; that is, from older screen-film mammography (SFM) or more recently from digital mammography.

Image quality differs between SFM and digital mammography—for example, in terms of object visibility and spatial resolution [11]—and thus a reader’s assessment of threshold-based MD may also differ between these modalities. Further, digital images are acquired in a raw (‘for processing’) format, in which the greyscale is proportional to X-ray attenuation. The processed (‘for presentation’) image is a manipulation of the raw image to aid tumour detection, based on manufacturer-specific algorithms which are generally unspecified and thus irreversible. Because processing may suppress or enhance image features such as dense tissue, MD measurements may systematically differ between the original raw and the processed images. The raw image is often deleted and only a processed format is available for MD measurements. Further, differences in MD between raw and processed images may vary by the type of digital mammography; that is, computed radiography (CR, a digital extension of screen film) or direct digital.

Two previous studies of MD in raw–processed pairs showed different results. From a General Electric (GE) Senographe 2000D model, percent MD (PMD) was higher in processed than in raw images [12]; whereas on images captured on a GE Senographe DS model [10], PMD was lower in processed than in raw images for one reader, but not different for another reader. We are not aware of raw–processed MD comparisons for other mammography systems.

In the present study, we extended the examination of MD across three widely used digital mammography systems (GE and Hologic, both direct digital, and Fuji, a CR system) by comparing threshold-based MD measurements for the same mammogram saved in both raw and processed formats and estimating MD conversion equations between these formats. In a similar fashion, we examined differences in MD between digitized SFM and processed CR-digital images taken from the same woman during consecutive screening rounds.

## Methods

### Source of images

For raw–processed MD comparisons, we included women who had both raw and processed image pairs available; that is, the same mammogram from a single screening session was saved in both formats. To examine different digital mammography system manufacturers (hereafter ‘systems’) we acquired six sets from three systems (Table 1): two direct digital systems (Hologic: sets H1, H2 and H3; and GE: sets G1 and G2) and a Fuji CR system (set F1). Hologic images were all captured on Lorad Selenia models whereas the GE images were captured on different models; Senographe 2000D, DS and Essential. Image sets originated from the Chilean Cohort Study of Breast Cancer Risk [13] (set H1), the Bahcesehir Mammographic Screening project in Turkey [14] (set H2), screening mammograms from the H. Lee Moffitt Cancer Center, Florida, USA (sets H3 and G1) [12] and the East London Breast Screening Programme, UK (set G2) [7]. These five sets reflect populations with nearly 3-fold differences in BC incidence rates [15]. In contrast, set F1 is a pooled resource of anonymized Fuji CR images taken for 100 women in 2008, on which both right craniocaudal (CC) and left CC images were saved in both formats (400 images). Other than age for 47 women, no other information was known about these women﻿. Thus whilst all other sets were from BC-free women, we cannot guarantee this status for set F1. All mammograms were taken between 2007 and 2013. Two sets, H1 and G2, also contributed to the International Consortium on Mammographic Density (ICMD) [16].

**Table 1:** Characteristics of mammograms and of women with raw–processed image pairs and SFM–digital image pairs

	Raw–processed image pairs						Processed digital–SFM pairs
	Set H1	Set H2	Set H3	Set G1	Set G2	Set F1	Set F2
Mammography system	Hologic (DD)			GE Medical Systems (DD)		Fuji (CR)	Fuji (SFM and CR)
Mammography machine	Lorad Selenia			Senographe 2000D	Senographe Essential (152 pairs), Senographe DS (87 pairs)	Clearview CSm	–
Views	L MLO	L MLO	L CC or R CC	L CC or R CC	L MLO	L CC and R CC	R CC
Pixel size (μm)	70	70	NK	NK	94 (91%), 100 (9%)	50	SFM: 50 (33%), 200 (67%); CR 50 (50%), 100 (50%)
Processing software version	AWS 3_3_1	AWS 3_4_1	NK	NK	ADS_43.10.1 (34.2%), ADS_54.10 (56.9%), ADS_54.11 (8.9%)		
Number of image pairs	186	73	417	180	238	200	139
Number of women	186	73	417	180	238	100	139
Source of films	Chilean Cohort Study of Breast Cancer Risk (in ICMD)	Bahcesehir screening programme, Turkey	H. Lee Moffitt Cancer Centre, USA	H. Lee Moffitt Cancer Centre, USA	East London Breast Screening Centre, UK (in ICMD)	NK	BreastScreen Victoria, Australia
Year^a^	2011–2013	2010–2011	2008–2010	2007–2011	2010–2012	2008	2004–2009
Age^a^ (years), mean (SD)	41.0 (4.4)	49.5 (7.5)	63.5 (10.7)	58.5 (10.4)	58.0 (5.8)	55.1 (12.8)^b^	57.9 (5.1) first screen, 60.0 (5.1) second
BMI^c^ (kg/m^2^), median (IQR)	27.6 (24.9–32.1)	NK	27.6 (24.3–32.4)	24.7 (22.3–27.0)	24.6 (22.5–28.8)	NK	NK

^a^At the time of mammography

^b^Age was known for 47 of 100 women only. Set F1: both R CC and L CC images were saved in raw and processed formats, therefore there are 100 women and 200 image pairs

^c^BMI at or near to mammography

*L* left, *R* right, *CC* craniocaudal, *MLO* mediolateral oblique, *GE* General Electric, *SFM* screen-film mammography, *DD* direct digital, *CR* computed radiography, *IMCD* International Consortium on Mammographic Density, *IQR* interquartile range, *NK* not known, *SD* standard deviation

For the comparison of MD assessed on SFM and digital mammography (Table 1, set F2, BreastScreen Victoria, Australia), we obtained pairs of view and laterality-matched films for the same 139 woman who were screened on SFM at one screening round and on a digital CR Fuji system at the next, a median of 2.1 years later (range 1.2–2.5 years).

Ethics approvals were obtain from IARC (IEC 12–34 for the ICMD) and from contributing studies.

### MD measurements

To improve readability of raw images, greyscale levels were transformed using a log-inversion implemented in Niftyview [17]. This process creates a ‘positive’ image out of the raw ‘negative’ and restores the approximately linear relationship between image intensity and tissue density exhibited by SFM. MD was measured in Cumulus version 3 or 6, in which the reader selects the threshold to dichotomize dense and non-dense pixels. These versions give equivalent MD measurements, but differ in ease of use for the reader. Measures obtained are areas (cm^2^) of the breast, the dense area (DA) and the non-dense area, and PMD, calculated as:

$$ \mathrm{P}\mathrm{M}\mathrm{D} = 100 \times \mathrm{D}\mathrm{A}\ /\ \mathrm{breast}\ \mathrm{area}. $$

Image sets were read by four experienced readers (VAM, Id-S-S, NFB and JH) in combinations dependent on permissions for inter-institutional image transfers. Sets H1, H2 and G2 were distributed randomly into 12 batches of 100 images (six raw and six processed batches) and allocated randomly to three readers. Each pair was read by the same reader. Each batch included three within-batch repeats and five images from each batch were repeated in the other two readers’ batches. The Fuji images (F1) and the SFM-digital image set (F2) were mainly read by a single reader. Sets H3 and G1 were not transferred between institutions, but had been measured previously by one reader as published previously [12].

Twelve image pairs were excluded because one or both images were indicated for exclusion upon MD measurement (e.g. due to low image quality, breast implants).

### Statistical methods

The primary outcome is PMD (%), and secondary outcomes are DA and breast area. For each of these, we used a square-root transformation (e.g. √PMD) to normalize distributions [18]. The interpretation of these measures can be aided by considering each area as a square, thus √DA and √breast area are the width in centimetres of the square. Similarly, √PMD can be thought of as the width of the dense square for a 10 cm × 10 cm breast area.

For each image format, within-reader reliability of √MD was assessed using the intraclass correlation coefficient:

$$ {{\mathrm{ICC} = \upsigma}^2}_{\mathrm{b}}/\left({\upsigma^2}_{\mathrm{b}}{{ + \upsigma}^2}_{\mathrm{w}}\right). $$

Between-women variance (σ^2^_b_) and within-reader variance (σ^2^_w_) were estimated in ANOVA models fitted on sets H1, H2, G2, F1 and F2 and all of the ICMD measurements combined. Sets H3 and G1 did not have within-reader repeats.

To estimate within-pair raw–processed differences in MD, we fitted multi-level normal-error regression models of √MD, where the fixed effect of image format was level 1 and a random intercept for woman was level 2. The assumption of a constant difference in √MD across the MD range was examined using Bland–Altman plots. Subgroup analyses were conducted by reader, system, model and processing software version, and by PMD and breast area categories and possible effect modification tested using likelihood ratio tests. These potential effect modifiers are features of the image or of the imaging process; woman-level characteristics such as body mass index (BMI) or age were not investigated, because potential effect modification would be mediated through image characteristics.

A similar approach was used to compare SFM and digital processed images for set F2.

Calibration equations for conversion between MD measured on raw and processed images, and vice versa, were based on √DA because all √PMD differences were driven through √DA whilst the change in √breast area was negligible (<1 mm). Standard regression models were not used as they assume error only in the dependent variable, which results in a fitted model that is not reversible (i.e. predicting raw from processed would give a different outcome to predicting processed from raw). Because there is measurement error in MD assessment on both raw and processed films, we applied a reversible conversion method. The principle of this calibration method was to maintain, for each reader and system combination, equality of the standard normal *z* scores of √DA whether they were assessed on a processed image (*z*_p_) or a raw image (*z*_r_):

$$ {z}_p=\left(\surd D{A}_p{\textstyle \hbox{-} }{\overline{x}}_p\right)/{s}_p $$

$$ {z}_r=\left(\surd D{A}_r{\textstyle \hbox{-} }{\overline{x}}_r\right)/{s}_r, $$

where *x̄* and *s* are the mean and standard deviation for the image type respectively. This method yields the following conversion equation:

$$ \surd D{A}_r={\overline{x}}_r+{s}_r{z}_p. $$

## Results

**Fig. 1:**
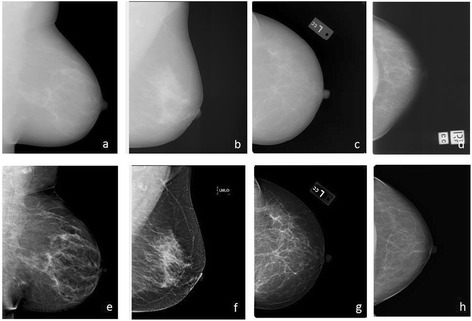
Examples of raw and processed images from Hologic, GE and Fuji digital mammography systems. **a** Raw and **e** processed paired images captured on GE Senographe Essential (G2, UK). **b** Raw and **f** processed paired images captured on Hologic Lorad Selenia (H1, Chile). **c** Raw and **g** processed paired images captured on Fuji CR (F1). **d** Screen-film image and **h** its paired Fujifilm CR processed image (SFM/digital set F2, Australia). *CC* craniocaudal, *L* left, *MLO* mediolateral oblique, *R* right

In total, 1294 raw–processed digital image pairs (2588 images) were analysed: 676 pairs captured on Hologic Lorad Selenia direct digital systems (CC and mediolateral oblique (MLO)), 418 on GE Senographe direct digital systems (CC and MLO) and 200 from Fuji CR (CC only) (Table 1). For digital image pairs, women were aged from 26 to 87 years at mammography (mean 55.1, SD 12.8) and the median BMI was 26.2 kg/m^2^ but varied between sets. Median overall PMD ranged between 15.4 and 24.8% and median DA ranged between 23.6 and 30.4 cm^2^ (Table 2) and reader-specific median measures are given in (Additional file 1: Table S1). Visual examination of sample raw–processed image pairs shows different degrees of accentuation of breast features and of the skin edge (Fig. 1).

**Table 2:** Percent density, dense area and total breast area in raw–processed image pairs and in SFM–processed digital image pairs

		Raw–processed image pairs				SFM–digital
		Hologic	GE	Fuji	All	Fuji CR
Number of women		676	418	100	1194	128
Number of image pairs		676	418	200	1294	128
Number of image pairs by view	L MLO	259	238	0	497	
	L CC	208	79	100	387	
	R CC	209	101	100	410	128
	All	676	418	200	1294	128
Number of potential MD readings (including 22% repeats), by reader	Reader 1	234	232	60	526	0
	Reader 2	246	218	60	524	0
	Reader 3	232	222	460	914	283
	Reader 4	834	360	0	1194	0
	All	1546	1032	580	3158	283
PMD^a^ (%)	Raw	15.4 (6.7–27.7)	18.5 (8.5–32)	23.1 (12.5–34.3)	18.1 (8.6–30.5)	SFM: 22.2 (15.6–28.5)
	Processed	18.7 (11.4–27.9)	21.8 (11.3–35.7)	24.8 (13.4–36.6)	20.2 (11.7–31.7)	18.9 (13.0–26.9)
Dense area^a^ (cm^2^)	Raw	23.6 (12.1–41.3)	25.0 (11.7–40.3)	28.8 (19.9–45.3)	25.4 (13.5–41.7)	SFM: 32.4 (22.4–43.2)
	Processed	28.2 (19–41.9)	27.6 (16.1–47.6)	30.4 (20.3–50.7)	28.5 (18.2–44.8)	28.9 (20.3–38.1)
Breast area^a^ (cm^2^)	Raw	166.9 (127.9–216.1)	138.4 (108.4–173.1)	152.9 (111.4–207.1)	155.8 (116.9–201.3)	SFM: 154.4 (119.1–193.1)
	Processed	167.3 (127.5–214.4)	140.1 (109.9–175)	150.7 (112.7–206.2)	156.1 (117.3–201.5)	156.7 (122.7–202.1)

^a^Median (interquartile range)

*L* left, *R* right, *CC* craniocaudal, *MLO* mediolateral oblique, *GE* General Electric, *SFM* screen-film mammography, *CR* computed radiography, *PMD* percent mammographic density assessed in Cumulus version 6

Within-reader reliability of PMD was slightly higher in SFM (ICC 0.94, 95% confidence interval (CI): 0.93, 0.95) than in raw digital (ICC 0.91, 95% CI: 0.89, 0.93) or processed digital (ICC 0.89, 95% CI: 0.88, 0.91) images. This difference generally held across readers (Table 3) and was driven by higher within-reader repeatability from SFM than when measuring from digital images. In contrast, whilst readers 1 and 3 had higher ICCs for PMD and DA assessed on raw images than on processed images, this was reversed for reader 2. Reader 1 ICCs for PMD and DA did not differ between image formats for the Fuji CR or Hologic systems, whereas for GE images the ICCs were lower on processed than on raw images. Throughout, ICCs for PMD predominantly reflected those for DA because breast area ICCs were near 100% for all image formats, readers and systems (Table 3). Based on the subset of images that were read by all readers, mean raw-processed MD measures and correlation coefficients by reader are given in Additional file 2: Table S2 and correlations between readers by image type in (and Additional File 3: Table S3.

**Table 3:** Intra-class correlation coefficient, within-reader and between-woman SD of MD measures to assess repeatability of MD readings, by image format

	SFM						Raw digital						Processed digital					
Measure subset	N obs	N women	N repeats	ICC	Within-reader SD	Between-women SD	N obs	N women	N repeats	ICC	Within-reader SD	Between-women SD	N obs	N women	N repeats	ICC	Within-reader SD	Between-women SD
Percent mammographic density^a^																		
All	6659	6418	241	0.94	0.42	1.61	1243	1098	145	0.91	0.51	1.64	5009	4627	394	0.89	0.49	1.40
Reader 1 (H1, H2, G2, F1)	1886	1818	68	0.96	0.38	1.90	346	298	48	0.97	0.33	1.87	1539	1413	126	0.91	0.46	1.51
Reader 2 (H1, H2, G2, F1)	2464	2381	83	0.92	0.46	1.55	356	309	47	0.79	0.63	1.24	1545	1430	119	0.88	0.48	1.32
Reader 3 (H1, H2, G2, F1)	2309	2217	92	0.89	0.41	1.17	541	489	52	0.87	0.53	1.39	1925	1775	150	0.86	0.52	1.31
Hologic^b^ (H1, H2)							363	316	47	0.86	0.59	1.48	2742	2517	225	0.87	0.48	1.25
GE^b^ (G2)							590	536	54	0.92	0.54	1.77	1234	1146	88	0.87	0.59	1.49
Fuji^b^ (F1)							290	244	46	0.94	0.39	1.47	1033	951	82	0.94	0.39	1.52
Dense area^a^																		
All sets, all readers	6842	6589	253	0.94	0.48	1.82	1244	1099	145	0.88	0.71	1.95	5021	4616	393	0.85	0.67	1.59
Reader 1 (H1, H2, G2, F1)	1963	1888	75	0.95	0.45	2.06	346	298	48	0.97	0.40	2.12	1543	1417	126	0.89	0.59	1.66
Reader 2 (H1, H2, G2, F1)	2568	2482	86	0.93	0.49	1.81	357	310	47	0.71	0.89	1.39	1549	1426	119	0.85	0.64	1.50
Reader 3 (H1, H2, G2, F1)	2311	2217	94	0.89	0.48	1.41	541	489	52	0.84	0.75	1.72	1929	1778	151	0.80	0.75	1.47
Hologic^b^ (H1, H2)							363	316	47	0.84	0.86	1.94	2745	2520	225	0.83	0.64	1.43
GE^b^ (G2)							591	537	54	0.86	0.75	1.87	1243	1154	89	0.77	0.85	1.53
Fuji^b^ (F1)							290	244	46	0.94	0.44	1.82	1033	951	82	0.94	0.50	1.95
Breast area^a^																		
All	6597	6357	240	1.00	0.14	2.46	1243	1098	145	1.00	0.15	2.53	5009	4616	393	1.00	0.11	2.76
Reader 1 (H1, H2, G2, F1)	1873	1805	68	1.00	0.10	2.46	346	298	48	1.00	0.06	2.49	1539	1413	126	1.00	0.07	2.74
Reader 2 (H1, H2, G2, F1)	2442	2359	83	0.99	0.18	2.47	356	309	47	1.00	0.17	2.48	1545	1426	119	1.00	0.10	2.77
Reader 3 (H1, H2, G2, F1)	2282	2191	91	1.00	0.12	2.46	541	489	52	1.00	0.18	2.59	1925	1775	150	1.00	0.13	2.76
Hologic^b^ (H1, H2)							363	316	47	1.00	0.08	2.13	2742	2517	225	1.00	0.09	2.54
GE^b^ (G2)							590	536	54	0.99	0.19	2.46	1234	1146	88	1.00	0.09	2.53
Fuji^b^ (F1)							290	244	46	1.00	0.15	2.73	1033	951	82	1.00	0.15	3.01

Analysis: ICCs, within-reader SD and between-women SD were estimated from a one-way ANOVA using all ICMD measurements and sets H1, H2, G2, F1 and F2. Number of repeats is the number of images read at least twice, by the same or different readers. Reader 4 does not appear here because no repeated readings were available for this reader

Numbers of observations vary by MD measure because only dense area was measured if the breast edge was not visible, and only percent mammographic density if the pixel size was unknown

^a^Analysed on a square-root scale

^b^Within reader, within image type

*Obs* observations, *SFM* Screen-film mammography, *ICC* Intra-class correlation coefficient, *MD* Mammographic density, *SD* Standard deviation, *N* number of, *GE* General Electric

For processed–raw digital image pairs, the median PMD was higher when measured on processed images than on raw images, by 1.7–3.3 absolute percentage points depending on the system (Table 2). Similarly, the median DA was larger by 1.6–4.6 cm^2^, whereas the median breast area was similar. Regression results were similar: √PMD was 0.34 cm (95% CI: 0.28, 0.40) larger in processed images than in raw images, whilst √DA was 0.44 cm (95% CI: 0.36, 0.52) larger and √breast area did not differ (0.01 cm; 95% CI: −0.01, 0.02) (Table 4). These differences in PMD were approximately one-fifth of the between-women SD (Table 3). For a given reader, PMD and DA differences varied in magnitude between systems (heterogeneity *p* < 0.01 for readers 1–3, *p* = 0.21 for reader 4), and for a given system the differences varied in both magnitude and direction between readers (*p* < 0.001 for each system). Specifically, for readers 1, 3 and 4, √PMD was larger in processed than in raw images by 0.4–0.9 cm (reader 1), 0.1–0.7 cm (reader 3) and 0.4–0.6 cm (reader 4), depending on the system. In contrast, √PMD in processed compared with raw images for reader 2 was either not different (GE) or was smaller (Fuji CR system and Hologic). Mean √DA from processed images was 0.9 (95% CI: 0.7, 1.1) higher for reader 2 and 0.9 (95% CI: 0.7, 1.1) higher for reader 3 compared with reader 1. Between-reader differences were larger for raw images; mean √DA was 2.3 (95% CI: 1.9, 2.8) higher for reader 2 and 1.9 (95% CI: 1.4, 2.3) higher for reader 3 compared with reader 1. For SFM, between-reader differences were slightly smaller; mean √DA was 1.3 (95% CI: 1.1, 1.4) higher for reader 2 and 0.7 (95% CI: 0.5, 0.8) higher for reader 3 compared with reader 1. Breast area differences also varied between system–reader combinations, but average differences were extremely small in magnitude (<1.2 mm √breast area). Differences by model or processing software within a system were not significant (data not shown). Effect modification of DA and PMD differences by categories of PMD or of breast area (categories defined by the raw image) were significant (*p* < 0.001 for both). The differences tended to decrease with increasing PMD, but they increased with increasing breast area (Additional File 4: Table S4).

**Table 4:** Mean differences in MD measures between processed images and the corresponding raw digital image, by reader and mammography system

Reader	system	Number of images	Number of women	Percent density		Dense area		Breast area	
				Difference^a^ √PMD (95% CI)		Difference^a^ √Dense area (cm) (95% CI)		Difference^a^ √Breast area (cm) (95% CI)	
Reader 1									
	Hologic	234	104	0.91	(0.74, 1.08)	1.17	(0.96, 1.39)	0.01	(−0.03, 0.05)
	GE	232	98	0.62	(0.44, 0.80)	0.79	(0.57, 1.00)	0.09	(0.07, 0.11)
	Fuji	60	15	0.40	(0.20, 0.61)	0.51	(0.26, 0.75)	−0.12	(−0.17, −0.08)
	All	526	217	0.72	(0.61, 0.84)	0.93	(0.79, 1.06)	0.03	(−0.08, 0.84)
	*p* for heterogeneity^b^				0.007		0.003		<0.001
Reader 2									
	Hologic	246	109	−0.47	(−0.64, −0.30)	−0.60	(−0.85, −0.34)	0.05	(0.01, 0.09)
	GE	218	95	0.05	(−0.12, 0.23)	0.07	(−0.15, 0.30)	0.11	(0.07, 0.16)
	Fuji	60	15	−0.76	(−1.03, −0.48)	−0.92	(−1.27, −0.57)	0.06	(−0.01, 0.12)
	All	524	219	−0.28	(−0.40, −0.17)	−0.36	(−0.52, −0.19)	0.08	(0.05, 0.11)
	*p* for heterogeneity^b^				<0.001		<0.001		0.09
Reader 3									
	Hologic	232	98	0.10	(−0.04, 0.24)	0.12	(−0.07, 0.31)	0.01	(−0.03, 0.04)
	GE	222	95	0.69	(0.52, 0.85)	0.88	(0.64, 1.12)	0.00	(−0.03, 0.04)
	Fuji	460	200	0.10	(−0.02, 0.23)	0.13	(−0.03, 0.29)	0.03	(−0.01, 0.08)
	All	914	392	0.24	(0.16, 0.33)	0.31	(0.20, 0.43)	0.02	(0.00, 0.04)
	*p* for heterogeneity^b^				<0.001		<0.001		0.48
Reader 4									
	Hologic	834	417	0.55	(0.44, 0.65)	0.74	(0.60, 0.89)	−0.09	(−0.10, −0.08)
	GE	360	180	0.43	(0.28, 0.58)	0.50	(0.34, 0.67)	0.08	(0.01, 0.16)
	All	1194	597	0.51	(0.43, 0.60)	0.67	(0.56, 0.78)	−0.04	(−0.07, −0.02)
	*p* for heterogeneity^b^				0.21		0.056		<0.001
All readers combined									
	Hologic	1546	679	0.37	(0.29, 0.45)	0.50	(0.39, 0.61)	−0.04	(−0.05, −0.03)
	GE	1032	418	0.45	(0.34, 0.56)	0.56	(0.42, 0.69)	0.07	(0.04, 0.10)
	Fuji	580	200	0.04	(−0.09, 0.18)	0.06	(−0.10, 0.23)	0.02	(−0.03, 0.07)
	All	3158	1297	0.34	(0.28, 0.40)	0.44	(0.36, 0.52)	0.01	(−0.01, 0.02)
	*p* for heterogeneity^b^				<0.001		<0.001		<0.001

*p* for heterogeneity <0.001 between readers for each of the Hologic, GE and Fuji systems, for both percent density and dense area. For breast area, *p* for heterogeneity <0.001 also between readers on the Hologic system, and no difference between readers for breast area was found for GE (*p* = 0.07) and Fuji (*p* = 0.08)

^a^Differences are processed–raw images

^b^ *p* value for heterogeneity between systems, for a given reader

*CI* confidence interval, *MD* Mammographic density, *GE* General Electric, *PMD* percent mammographic density

**Fig. 2:**
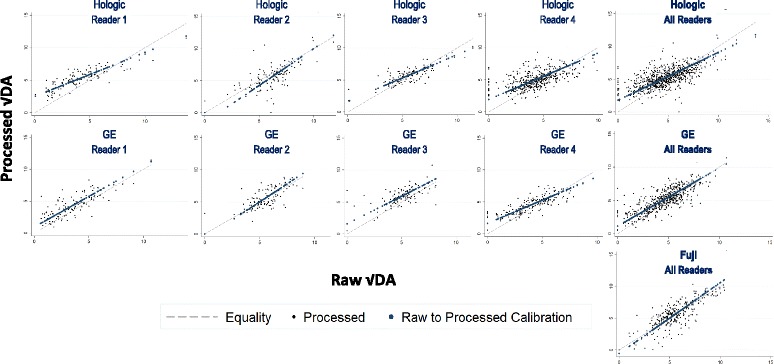
Scatter plot of paired √DA readings measured on processed (*y* axis) vs raw (*x* axis) digital images, by reader and system. *Dashed lines*, equality (if DA from processed images was read identically to raw images); *blue dots*, modelled linear conversion. Reader-specific and system-specific calibration equations for the conversion of raw √DA to processed √DA are supplied in (Additional file 7: Information 2). *√DA* square root of dense area, *GE* General Electric

Most scatter plots (Fig. 2) showed that differences in DA on processed images compared with raw images are larger at lower DAs, and converge towards no difference in breasts with a √DA of ≥5 cm. Bland–Altman plots also revealed that processed–raw differences in √PMD and √DA (Additional File 5: Figure S1) were not constant across the underlying MD range. However differences were constant on the standardized scale (shown for DA in Additional File 6: Figure S2), and thus calibration equations were based on standardized values of DA in the two image types. Figure 2 (Additional file 7: Information 1) presents these reader-specific and system-specific calibration equations for DA. Differences were very small for the Fuji CR and were larger and of a similar magnitude between the direct digital systems. For all readers combined, conversion equations from raw DA to their processed equivalent are as follows:

- Hologic: processed √DA = 5.252 + 0.719 (raw √DA – 4.751)
- GE: processed √DA = 5.081 + 0.872 (raw √DA – 4.523)
- Fuji: processed √DA = 5.694 + 1.107 (raw √DA – 5.633)

After correcting DA, the corrected non-dense area and PMD would then be calculated using the original breast area and preserving the original definitions:

$$ \mathrm{N}\mathrm{o}\mathrm{n}{\textstyle \hbox{-}}\mathrm{dense}\ {\mathrm{area}}_{\mathrm{c}}=\mathrm{breast}\ \mathrm{area}{\textstyle\ \hbox{-}\ }{\mathrm{DA}}_{\mathrm{c}} $$

$$ {\mathrm{PMD}}_{\mathrm{c}} = 100 \times {\mathrm{DA}}_{\mathrm{c}}/\ \mathrm{breast}\ \mathrm{area}. $$

Equations to generate √DA, as if measured on a raw image, from DA measured on a processed image are provided in (Additional file 7: Information 1).

For the processed–SFM set (F2), comparing MD measured on the processed digital image with that on the earlier SFM, √breast area was 0.17 cm larger (95% CI: 0.06, 0.28) and √DA was 0.17 cm smaller (95% CI: 0.01, 0.33).

## Discussion

### Findings

In the present study, we compared Cumulus-assessed MD measures (PMD, breast area and DA) on the same digital mammograms saved in processed and raw formats. Overall, we observed higher MD in the former image type, a difference that was not entirely consistent either in magnitude or direction across four readers for a given mammography system. Differences in MD assessed on raw and processed images were small for the CR system, but larger for direct digital systems. Differences between SFM and CR-digital images appeared to be small, although the latter were not time-matched comparisons. Readers had higher MD repeatability for SFM images than for raw or processed digital images. This may be because readers had more experience of reading from SFM images, or because density is more easily visualized in SFM images.

### Comparison and plausibility

Readers noted several appearance qualities of processed images that may affect the MD assessment, such as ‘thickened breast edge’ or ‘faded parenchyma’. Processing algorithms involve multiple steps designed to clarify the image, enhance suspected lesions and reduce noise—this noise may be dense tissue, therefore it has been hypothesized that density would be lower in processed images. However, this and similar studies generally found higher MD on processed images, particularly at lower density levels. Enhancement of light/dark transitions and accentuation of the breast edge may contribute to this increase. Differences in PMD were almost entirely driven by changes in the DA because breast area altered minimally. Our results are also consistent with those of Keller et al. [10], and Martin et al. [19], who reported that differences were highly reader dependent. Unsurprisingly, Vachon et al.’s results [12], which comprised 14% of our raw–processed pairs, also found that PMD was overestimated in less dense breasts in processed compared with raw GE images. Studies that compared MD using the BIRADS classification did not find differences by image type [20], but differences may be too small to be detected using a broad categorical classification.

Differences in MD assessment between SFM and Fuji CR were not assessed optimally, because they were based on films taken 2 years apart. While there was no breast area difference in the time-matched images, over this time interval the breast area increased indicating measurable age-related changes. The magnitude of this increase (0.17 cm √breast area) was consistent with the expected within-woman changes (0.16 cm over 2 years) found in a previous SFM-only longitudinal study [21]. Similarly, the decline in DA was only slightly larger than would be expected from age-related changes (−0.13 cm √DA), suggesting that any differences due to image formats were small (at most 0.04 cm). However, similar studies comparing PMD in SFM and digital mammography reported that PMD was higher in SFM images than in raw or processed digital images [22], including one in which the digital and the SFM were taken on the same day [19]. In both studies the differences were larger than for the present study, possibly because they were comparing SFM with direct digital and not with CR as in the present study. Breast area was also higher in digital images taken on the same day as SFM images, indicating that lower PMD assessment may be a product of both underestimation of DA and overestimation of breast area in digital images compared with SFM images. Harvey [22] hypothesized that more subcutaneous fat is included in digital measurements because the breast edge can be seen and delimited more precisely, but only PMD was reported in that study. In the present study, small differences between SFM and CR may reflect these closely related imaging technologies; CR systems are additions to SFM systems, using phosphor plates and a separate reader to create digital images, whereas the direct digital image is created at the point of image capture [23]. Thus, CR images have lower spatial resolution and more image noise than direct digital images [24]. The improved image quality in direct digital allows for more complex multi-functional processing algorithms, which may account for the larger raw–processed differences in direct digital images compared with CR images.

### Strengths and limitations

This is the first study to compare raw and processed images, using the same design and analytic approach, captured on several widely used mammography systems. Comparisons of MD across multiple systems are important because it is unlikely that all women in a study, or the same woman followed for several years, will be screened on the same mammography machine. Nevertheless, several design features would have improved the study; by including CC views alongside MLO for all images, and including other widely used mammography systems such as Siemens, and other CR systems. We were limited by the lack of information on manipulations performed by processing algorithms which are proprietary to manufacturers. Multiple readers are a further strength, being reflective of clinical and research settings—between-reader differences in raw–processed calibration highlight the need to recognize and quantify these differences where possible. Further, we used a reversible statistical method for processed–raw MD conversions; that is, neither raw nor processed MD is considered the error-free independent variable, which would not have been the case had a simple regression method been used. Finally, the women included in this study came from countries with a wide range of BC incidence rates, and thus the results should be generalizable to women across the BC risk spectrum.

### Relevance and implications

The potential impact of raw–processed differences in MD from direct-digital systems (3.3 percentage points) will depend on the application. When investigating MD as a predictor of BC risk, differences are unlikely to introduce substantial misclassification between very low density (<10%) and very high density (e.g. >50%) and would thus have a small impact on relative risk estimates. For investigations of determinants of MD or changes in MD, raw–processed differences are of a magnitude similar to 10 years of aging or the menopause-related PMD change (as assessed within ICMD) and depend greatly on the reader. Thus, in the screening or clinical setting when assessing MD change over time for the same woman, it is important that the same reader reads the woman’s repeat mammograms. If the calibration equations presented in this article are to be used in the screening or clinical settings, they will need to be validated, particularly for different readers. In studies comparing PMD across raw and processed image types, correcting for these differences is thus important and would ideally be made using reader-specific and system-specific calibrations. Even if all images are of the same type (raw or processed) it is necessary to calibrate between readers. Comparability of raw images between systems has not been assessed and difference in acquisition between systems may be present. The repeated finding across studies of large between-reader differences in MD, in addition to their time-intensive nature, again emphasizes the need for fully-automated methods of MD measurement. Four such fully automated quantitative methods were recently evaluated for BC risk prediction, alongside Cumulus [7]. Although such methods eliminate between-reader variations in readings, many only work on a single image type (often raw digital images [25]), but others can be applied across multiple types [8, 26]. It is possible that there would be between-system differences in automated measures, particularly volumetric measures due to differences in breast positioning and therefore breast thickness [27], but not all studies have found this [28]. In the future, as further processing algorithms are developed, MD differences between raw and processed images are likely not only to persist but also to change. However, as digital storage becomes cheaper and faster, such problems may be overcome if raw images are systematically stored and MD is consistently measured on them. In a similar fashion, a consistent and fully-automated MD measurement tool could be applied to the raw image bank to provide MD data in an efficient and systematic manner.

## Conclusion

Processed ‘for presentation’ direct digital mammograms have, on average, a higher Cumulus-assessed PMD and dense area compared with their corresponding raw ‘for processing’ images, whilst such differences were small for CR systems. Raw–processed differences in the direct digital systems depended on mammography system and to a large extent on reader, as did absolute density readings for a given image type. Controlling for these factors is necessary when comparing density readings across image types. For detection of small differences in density (e.g. within-woman changes), reader-specific processed to raw calibration, or restriction of comparisons to readings made by the same reader and on the same image type ﻿may be necessary.

## Additional files

Additional file 1: is **Table S1** presenting percent density, dense area and total breast area in raw–processed image pairs and in SFM–processed digital image pairs, by reader. (DOC 33 kb)
Additional file 2: is **Table S2** presenting mean MD measures of inter-reader repeats, by reader and image type. (DOC 29 kb)
Additional file 3: is **Table S3** presenting correlation of MD measures in inter-reader repeats. (DOC 29 kb)
Additional file 4: is **Table S4** presenting mean differences in MD measures between processed images and the corresponding raw digital image, by percent density and breast area categories. (DOC 30 kb)
Additional file 5: is **Figure S1** showing Bland–Altman plots for *v*MD measures, by mammography system and reader for: (**A**) percent mammographic density, (**B**) dense area and (**C**) breast area. *Y* axes to the same scale for comparisons. (DOCX 138 kb)
Additional file 6: is **Figure S2** showing Bland–Altman plots for within-system and within-reader standardized *v*DA measures. (DOCX 79 kb)
Additional file 7: is **Information 1** showing calibration equations for the conversion of raw *v*DA to processed *v*DA, and Information 2 showing calibration equations for the conversion of processed *v*DA to raw *v*DA. (DOC 41 kb)

## Abbreviations

BC: Breast cancer
BMI: Body mass index
CC: Craniocaudal
CI: Confidence interval
CR: Computed radiography
DA: Dense area
GE: General Electric
ICC: Intra-class correlation coefficient
ICMD: International Consortium on Mammographic Density
MD: Mammographic density
MLO: Mediolateral oblique
PMD: Percent mammographic density
SD: Standard deviation
SFM: Screen-film mammography
