# Supplementary material for: Mammographic density assessed on paired raw and processed digital images and on paired screen-film and digital images across three mammography systems
Source: Breast Cancer Res. 2016 Dec 19;18:130. doi: 10.1186/s13058-016-0787-0 (PMC5168805; doi:10.1186/s13058-016-0787-0)
Supplement: Additional file 1: — is Table S1 presenting percent density, dense area and total breast area in raw–processed image pairs and in SFM–processed digital image pairs, by reader. (DOC 33 kb) [file 13058_2016_787_MOESM1_ESM.doc]

**Additional file 1**

**Table S1: Percent density, dense area and total breast area in raw-processed image pairs and in SFM-processed digital image pairs, by reader**
