# Supplementary material for: Mammographic density assessed on paired raw and processed digital images and on paired screen-film and digital images across three mammography systems
Source: Breast Cancer Res. 2016 Dec 19;18:130. doi: 10.1186/s13058-016-0787-0 (PMC5168805; doi:10.1186/s13058-016-0787-0)
Supplement: Additional file 2: — is Table S2 presenting mean MD measures of inter-reader repeats, by reader and image type. (DOC 29 kb) [file 13058_2016_787_MOESM2_ESM.doc]

**Additional file 2**

**Table S2: Mean MD measure of inter-reader repeats, by reader and image type**
