# Supplementary material for: Mammographic density assessed on paired raw and processed digital images and on paired screen-film and digital images across three mammography systems
Source: Breast Cancer Res. 2016 Dec 19;18:130. doi: 10.1186/s13058-016-0787-0 (PMC5168805; doi:10.1186/s13058-016-0787-0)
Supplement: Additional file 3: — is Table S3 presenting correlation of MD measures in inter-reader repeats. (DOC 29 kb) [file 13058_2016_787_MOESM3_ESM.doc]

**Additional file 3**

**Table S3: Correlation of MD measures in inter-reader repeats**
