# Supplementary material for: Mammographic density assessed on paired raw and processed digital images and on paired screen-film and digital images across three mammography systems
Source: Breast Cancer Res. 2016 Dec 19;18:130. doi: 10.1186/s13058-016-0787-0 (PMC5168805; doi:10.1186/s13058-016-0787-0)
Supplement: Additional file 4: — is Table S4 presenting mean differences in MD measures between processed images and the corresponding raw digital image, by percent density and breast area categories. (DOC 30 kb) [file 13058_2016_787_MOESM4_ESM.doc]

**Additional file 4**

**Table S4: Mean differences in MD measures between processed and the corresponding raw digital image, by percent density and breast area categories**
