# Supplementary material for: Mammographic density assessed on paired raw and processed digital images and on paired screen-film and digital images across three mammography systems
Source: Breast Cancer Res. 2016 Dec 19;18:130. doi: 10.1186/s13058-016-0787-0 (PMC5168805; doi:10.1186/s13058-016-0787-0)

**Additional file 5**

**Figure S1: Bland-Altman plots for square-root MD measures, by mammography system and reader for:**

**(A) percent mammographic density, (B) dense area and (C) breast area. Y-axes are to the same scale for comparisons.**

**A) Square-root percent density**

**
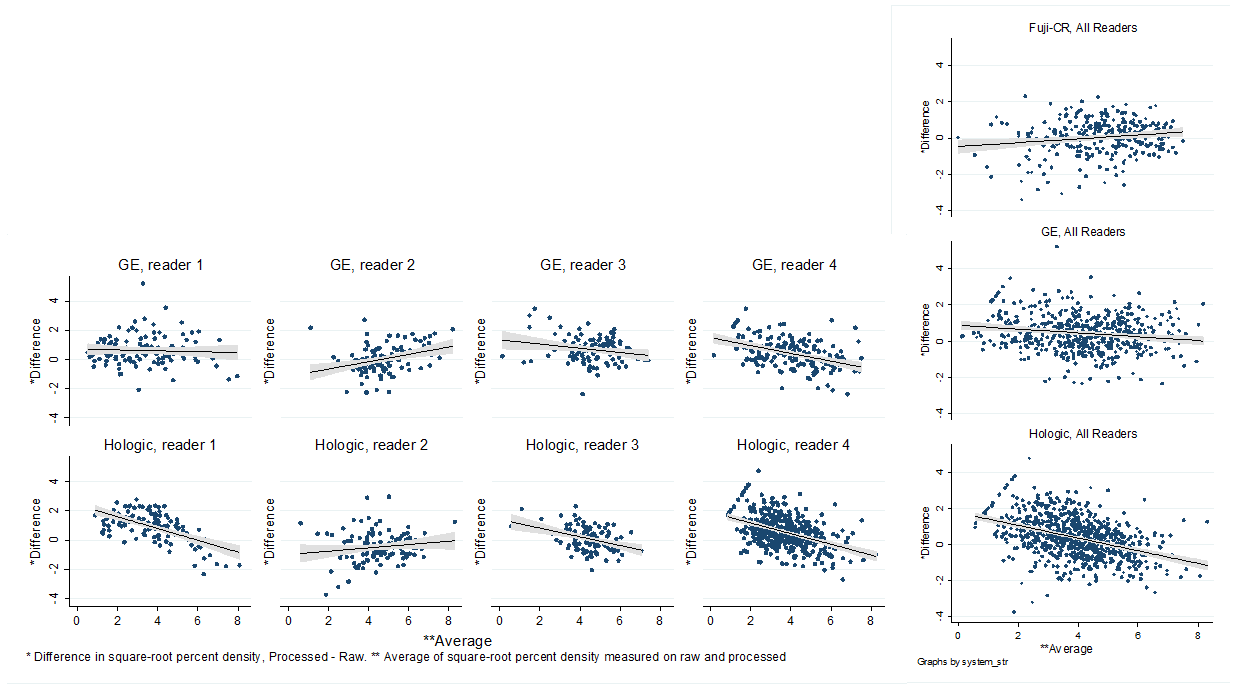
**

**B) Square-root dense area (cm)**

**
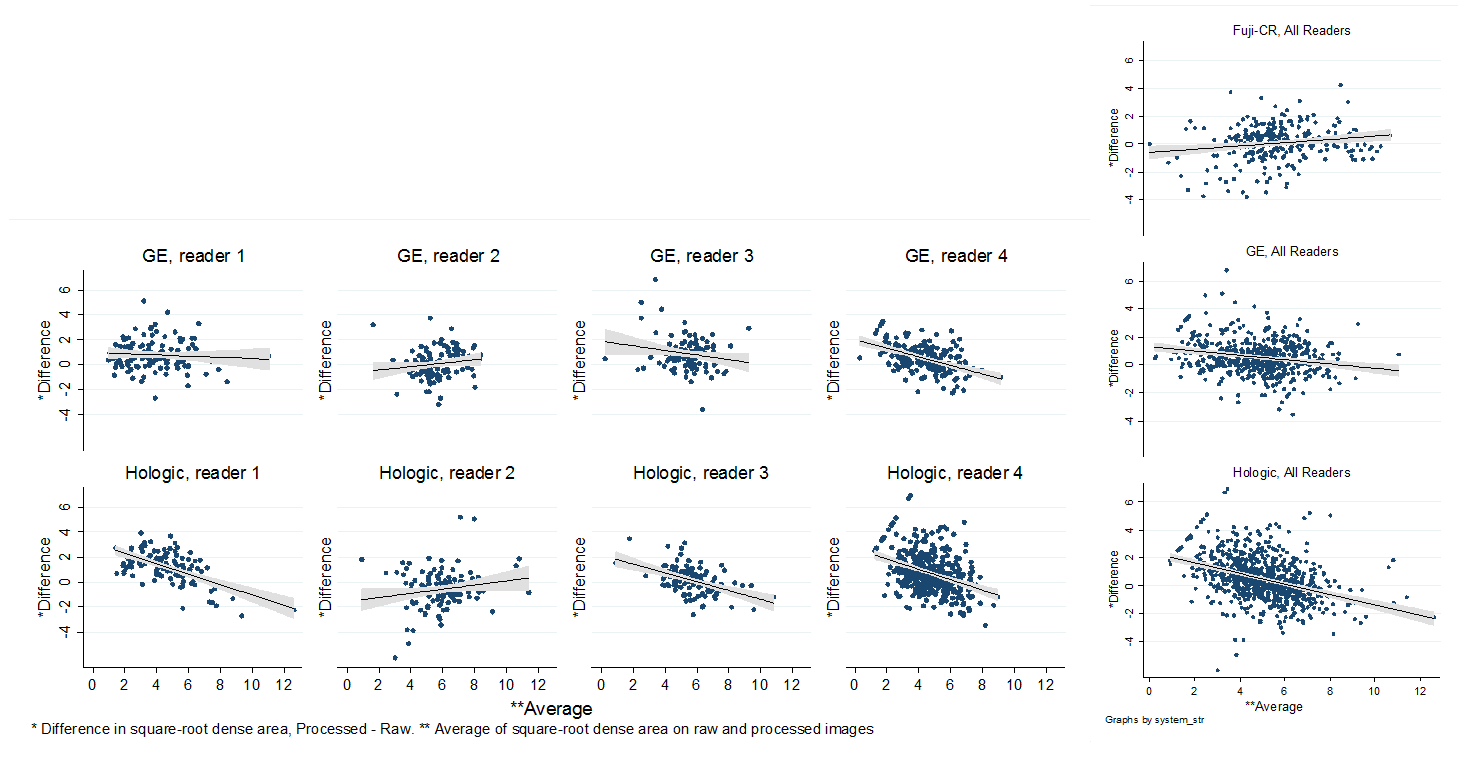
**

**C) Square-root breast area (cm)**


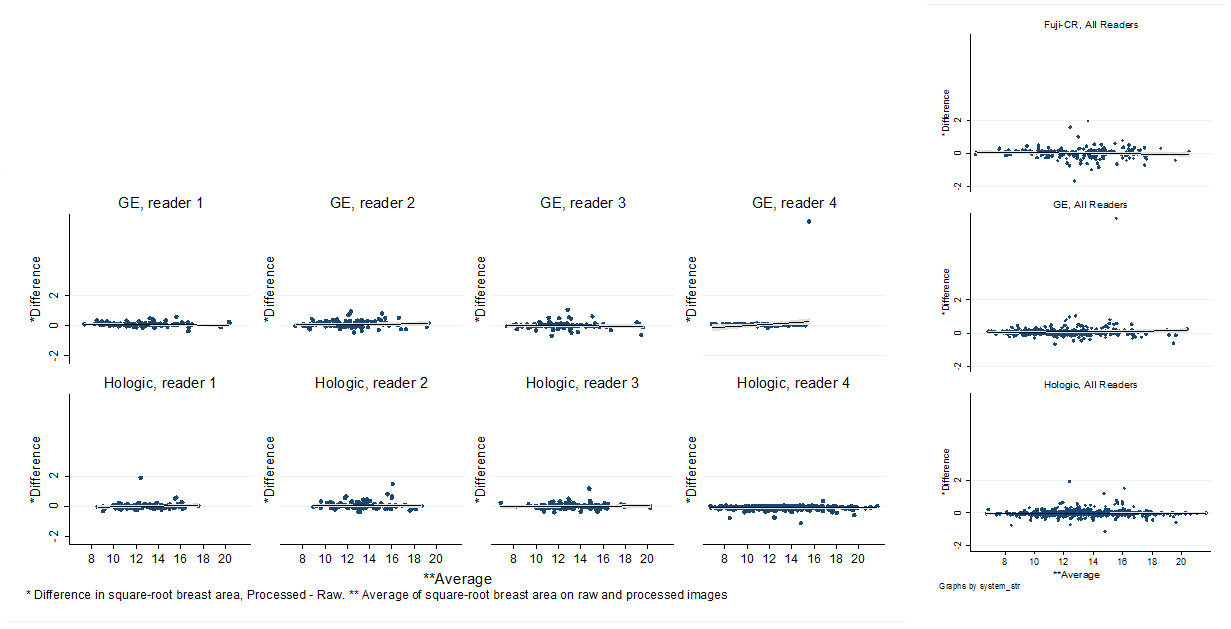

Supplement: Additional file 5: — is Figure S1 showing Bland–Altman plots for vMD measures, by mammography system and reader for: (A) percent mammographic density, (B) dense area and (C) breast area. Y axes to the same scale for comparisons. (DOCX 138 kb) [file 13058_2016_787_MOESM5_ESM.docx]
