# Supplementary material for: Mammographic density assessed on paired raw and processed digital images and on paired screen-film and digital images across three mammography systems
Source: Breast Cancer Res. 2016 Dec 19;18:130. doi: 10.1186/s13058-016-0787-0 (PMC5168805; doi:10.1186/s13058-016-0787-0)

**Additional file 6**

**Figure S2: Bland-Altman plots for within-system and reader standardized square-root dense area measures**


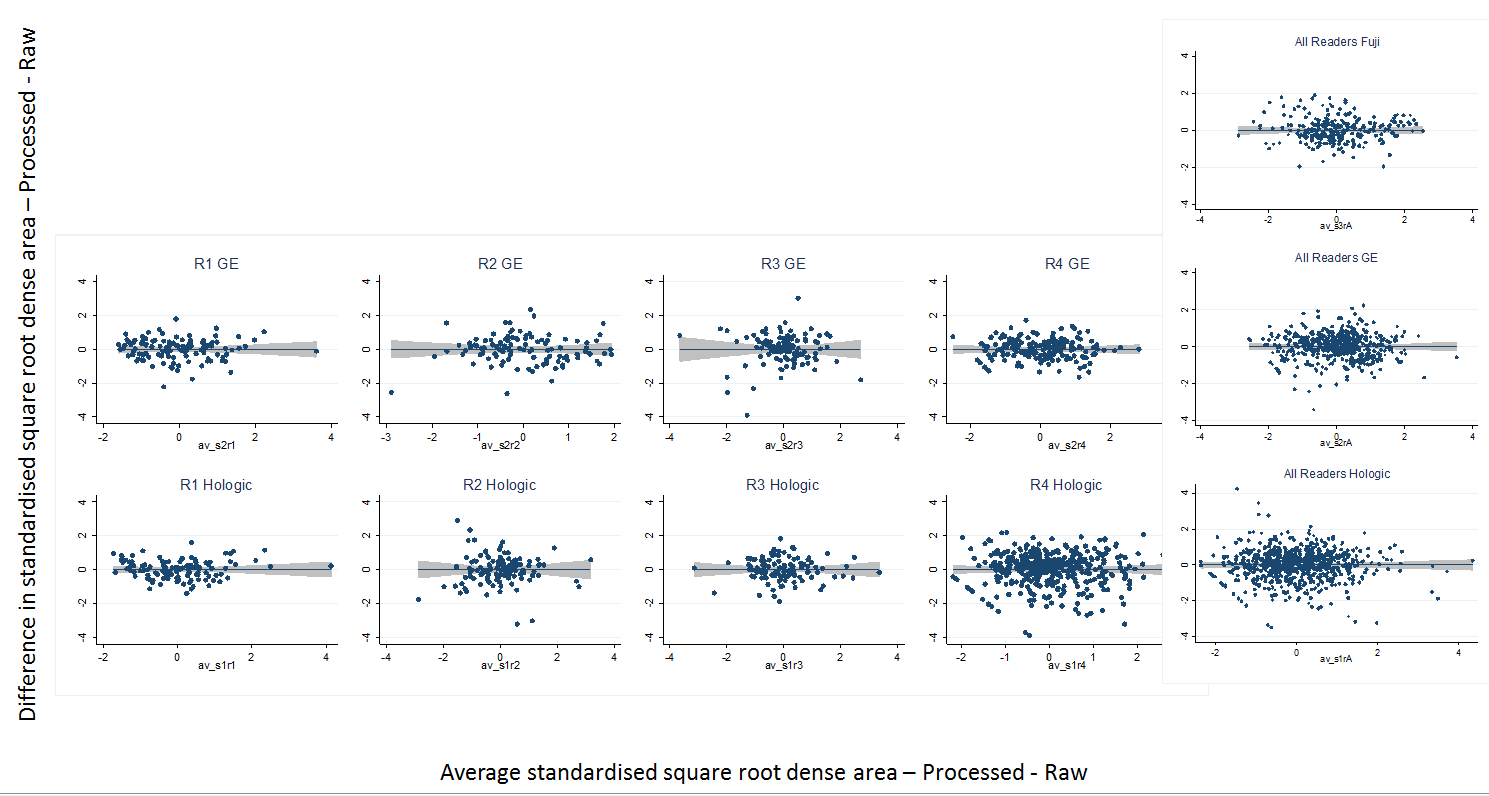

Supplement: Additional file 6: — is Figure S2 showing Bland–Altman plots for within-system and within-reader standardized vDA measures. (DOCX 79 kb) [file 13058_2016_787_MOESM6_ESM.docx]
