# Supplementary material for: Mammographic density assessed on paired raw and processed digital images and on paired screen-film and digital images across three mammography systems
Source: Breast Cancer Res. 2016 Dec 19;18:130. doi: 10.1186/s13058-016-0787-0 (PMC5168805; doi:10.1186/s13058-016-0787-0)
Supplement: Additional file 7: — is Information 1 showing calibration equations for the conversion of raw vDA to processed vDA, and Information 2 showing calibration equations for the conversion of processed vDA to raw vDA. (DOC 41 kb) [file 13058_2016_787_MOESM7_ESM.doc]

**Additional file 7**

**Information 1**

Calibration equations for the conversion of raw square root dense area (DA) to processed square root dense area:

| **System** | | **Reader** | **Raw to Processed Conversion Equation** |
| --- | --- | --- | --- |
| **Hologic** | 1 | | Processed √DA=5.246+((1.543/2.28) ×(Raw √DA-4.075)) |
|  | 2 | | Processed √DA=5.581+((1.882/1.641)×(Raw √DA-6.178)) |
|  | 3 | | Processed √DA=5.794+((1.291/1.78)×(Raw √DA-5.671)) |
|  | 4 | | Processed √DA=5.006+((1.28/1.777)×(Raw √DA-4.264)) |
|  | **All** | | **Processed √DA=5.252+((1.464/2.008)×(Raw √DA-4.751))** |
| **GE** | 1 | | Processed √DA=4.441+((1.895/1.979)×(Raw √DA-3.655)) |
|  | 2 | | Processed √DA=5.645+((1.501/1.337)×(Raw √DA-5.571)) |
|  | 3 | | Processed √DA=5.914+((1.34/1.553)×(Raw √DA-5.031)) |
|  | 4 | | Processed √DA=4.637+((1.429/1.963)×(Raw √DA-4.133)) |
|  | **All** | | **Processed √DA=5.081+((1.657/1.901)×(Raw √DA-4.523))** |
| **Fuji** | **All** | | **Processed √DA=5.694+((2.077/1.867)×(Raw √DA-5.633))** |

**Information 2**

Calibration equations for the conversion of processed square root dense area (DA) to raw square root dense area:

| **System** | **Processed to Raw Conversion Equations** |
| --- | --- |
| **Hologic** | Raw √DA = 4.751 + (2.008/1.464) × (Processed √DA-5.252) |
| **GE** | Raw √DA = 4.523 + (1.901/1.657) × (Processed √DA-5.081) |
| **Fuji** | Raw √DA = 5.633 + (1.876/2.077) × (Processed √DA-5.694) |
